# Feasibility study and pilot cluster-randomised controlled trial of the GoActive intervention aiming to promote physical activity among adolescents: outcomes and lessons learnt

**DOI:** 10.1136/bmjopen-2016-012335

**Published:** 2016-11-11

**Authors:** Kirsten Corder, Helen E Brown, Annie Schiff, Esther M F van Sluijs

**Affiliations:** MRC Epidemiology Unit, UKCRC Centre for Diet and Activity Research (CEDAR), University of Cambridge School of Clinical Medicine, Institute of Metabolic Science, Cambridge, UK

**Keywords:** physical activity, promotion, intervention, health behaviour, adolescent

## Abstract

**Objectives:**

Assess the feasibility of implementing the GoActive intervention in secondary schools, to identify improvements, test study procedures, determine preliminary effectiveness to increase moderate-to-vigorous physical activity (MVPA), and inform power calculations to establish programme effectiveness.

**Setting:**

Feasibility study (1 school) and pilot cluster-randomised controlled trial (CRCT; 2 intervention; 1 control school(s)).

**Participants:**

460 participants (46.6% female; 13.2 (0.4) years old).

**Interventions:**

8-week intervention (2013) involved: classes choosing weekly activities encouraged by mentors (older adolescents) and in-class peer leaders. Students gain points for trying activities which are entered into an intramural competition.

**Primary and secondary outcome measures:**

Planned quantitative (questionnaire) and qualitative (focus groups) process evaluation addressed enjoyment, confidence, participation, suggested improvements. Outcomes were assessed at baseline and follow-up (week 8) in pilot CRCT and included accelerometer-assessed MVPA; adolescent-reported activity type, well-being, peer support, shyness, sociability. Analysis of covariance was used to assess preliminary effectiveness as change in MVPA adjusted for baseline.

**Results:**

All year 9 students in intervention schools were exposed to the intervention; over all schools 77% of eligible students were measured. 71% boys and 74% girls found GoActive ‘fun’; 38% boys and 32% girls said it increased confidence, and 64% boys and 59% girls said they would continue with a GoActive activity. Suggested improvements included more mentorship; improved training; streamlined points recording. Pilot results indicated potential effectiveness ((adjusted mean difference (95% CI) p value; MVPA mins; 5.1 (1.1 to 9.2) p=0.014)) and suggest recruitment of 16 schools (2400 adolescents) for a full trial. Compared with control, intervention students reported greater peer support 0.5 (0.1 to 0.9) p=0.03, well-being 1.8 (0.1 to 3.4) p=0.04 but no difference in shyness/sociability. Participation in activity types approached significance (intervention group 2.3 (−0.2 to 4.7) p=0.07 more activity types).

**Conclusions:**

Results suggest feasibility and indicate potential effectiveness of GoActive to increase MVPA and support a fully powered evaluation of effectiveness and cost-effectiveness. Process evaluation data were used to refine GoActive prior to a full trial.

**Trial registration number:**

ISRCTN31583496; pre-results.

**Strengths and limitations of this study**

- We describe the feasibility and pilot testing of a health promotion intervention prior to a fully powered trial; this process follows the Medical Research Council (MRC) guidance for developing and evaluating complex interventions.
- It is important to use and publish feasibility and pilot research as often it is not properly used by researchers let alone published to enable use by others developing similar programmes. By combining feasibility, pilot results and lessons learnt in one paper, we are highlighting the most useful and salient messages without an excessive number of publications.
- These pilot cluster-randomised controlled trial results provide an indication of the potential effectiveness of GoActive to increase moderate-to-vigorous physical activity (min/day). However, there were not sufficient clusters to be able to adjust for school clustering in the analysis; results should therefore be interpreted with caution.
- We did not collect cost-effectiveness data in the feasibility and pilot studies and will put in place school-relevant mechanisms to collect the necessary data for an economic evaluation in the full trial.
- We collected valuable qualitative data during our participant and mentor focus groups but could not conduct formal qualitative analysis due to the need to progress the research at a timely pace, and to meet the timing of funding calls.

## Introduction

Most adolescents are insufficiently active^1^ ^2^ and this inactivity tracks into adulthood^3^ ^4^ increasing the risk of diabetes, cancer and mortality.^5^ ^6^ Pubertal, brain and social development during adolescence leads to new capacity for health behaviours^7^ increasing the likelihood of long-term behaviour change. In a meta-analysis of 30 physical activity (PA) intervention studies with objective outcomes,^8^ only two of the included studies focused on adolescents over the age of 13 years.^9^ ^10^ The 2012 UK Chief Medical Officers report states the importance of PA among young people,^11^ and the report from the UK All-Party Commission on Physical Activity calls the provision of a more diverse and inclusive offer of PA within schools.^12^ This highlights the lack of focus in this important group and an urgent need for the development and evaluation of potentially successful strategies.

We have previously described the development process of the GoActive intervention aiming to increase PA among 13–14 years old adolescents.^13^ This process included identifying gaps in the existing literature, large-scale quantitative adolescent opinion gathering,^1^ adolescent and teacher focus groups, adolescent interviews investigating engagement of the target group and development and refinement of the intervention.^13^ Feasibility and pilot testing of the GoActive programme is important to demonstrate intervention acceptability, feasibility of recruitment, randomisation and measurement of year 9 students. Data on preliminary effectiveness are also necessary to inform a realistic estimate of the resources needed for the evaluation of a fully powered randomised controlled trial. This work forms an integral part of a thorough development and evaluation process of PA promotion programmes for adolescents.^13^

We conducted a feasibility study of the GoActive intervention in one secondary school and a pilot cluster-randomised controlled trial (CRCT) in three schools (two intervention and one control; ISRCTN31583496).

In the feasibility study, we aimed to assess the feasibility of study recruitment and consent procedures and the implementation of the intervention across year 9.

The aim of the pilot CRCT was to assess preliminary effectiveness and to test full study procedures, including measurement logistics, randomisation and training of intervention facilitators outside of the research team. Further, having one control school allowed for estimation of preliminary effectiveness and of the number of participants required to adequately power a full trial. This process of feasibility and pilot testing prior to a full trial follows the Medical Research Council (MRC) guidance for developing and evaluating complex interventions.^14^

In this paper, we discuss the methods and results of the feasibility study which was conducted before the pilot CRCT. We then summarise improvements made to the intervention methods between the feasibility study and pilot CRCT. We then describe the methods and results of the pilot study including the suggestion of further changes required before a fully powered randomised controlled trial. Finally, an overall discussion gives an overview of the work as a whole.

## Feasibility study

The aim was to assess the feasibility of study recruitment and consent procedures and the implementation of the intervention across year 9.

## Methods

### School recruitment

Head teachers of all Cambridgeshire government-funded, all-ability, non-fee-paying (state) secondary schools within a 30 min drive of the study office were sent a letter inviting them to take part in a feasibility study to test an intervention aiming to increase PA among year 9 students. We conducted the feasibility study with the first school who agreed to participate (indicated by signing a school acceptance form). The school agreed to implement the GoActive intervention in the whole of year 9 and to allow us to conduct premeasurements and postmeasurements on consenting students, and was told that they would receive £200 of sports equipment for the school after completion of postintervention measurements.

### Participant recruitment

In the Summer term (April to July) of 2013 all year 9 students (n=234) and their parents at the participating school received invitation packs including study information and invitations for students to participate in preintervention and postintervention measurements. Parents were asked to provide passive consent (active opt-out consent) for their son/daughter to take part in the study measures. We gave parents at least 2 weeks to respond to this invitation and another copy of the letter was sent after 1 week. Parents were given the option to phone or email the study team if they did not consent for their child to take part in the study measures or they could complete a written opt-out form. Reminders and information about the study was additionally included in all relevant school media, including newsletters and emails and the usual reminders sent from the school. Written student assent was obtained by research assistants trained in good clinical practice prior to any measurements taking place.

### Intervention

**Figure 1:**
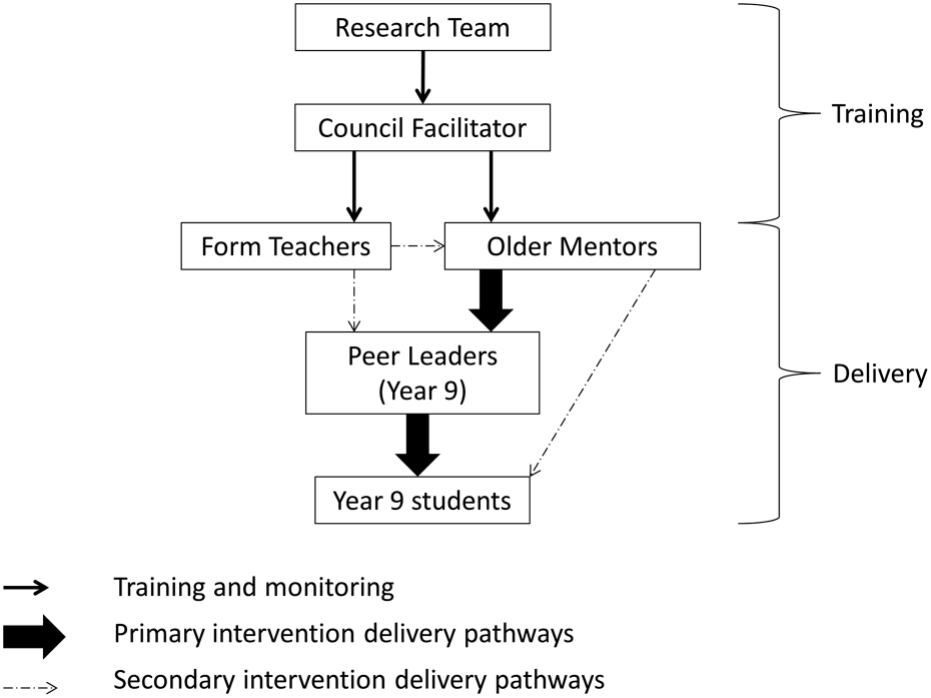
Tiered leadership system.

The GoActive intention has been described in detail previously,^13^ and the components are presented in table 1. Briefly, GoActive aims to increase PA through increased peer support, self-efficacy, self-esteem, group cohesion and friendship quality, and is implemented in tutor groups using a tiered leadership system (figure 1). Tutor groups choose two weekly activities each; mentors (older adolescents) and weekly peer leaders in each class encourage students to try these. Students gain points for trying new activities; points are entered into a between-class competition and weekly rewards are provided. Mentors and teachers support students to record and summarise their points. Mentors were to be given one training session by the study team and ongoing support by the intervention facilitators during the project. Teachers had a supportive role and were asked to encourage their class to participate and facilitate students to collect points.

**Table 1:** Description of the GoActive intervention according to key components

Concept	Component
Choice	Each tutor group chooses two different activities weekly.
Novelty	There are currently 20 activities available, designed to use little or no equipment and to be different from the usual school sports.
Mentorship	Older adolescents (mentors) are paired with each year 9 class encourage participation in activities. Mentors are helped by year 9 in-class peer leaders who change weekly.
Competition	Students gain points every time they do an activity; there is no time limit, students just have to try an activity to get points. Individual points are kept private with class level totals circulated to encourage interclass competition.
Rewards	Students gain small individual prizes for reaching certain points levels.
Flexibility	At least one tutor time weekly is used to do an activity and participants are also encouraged to do activities at other times, including out of school.

Tutor groups usually meet at the beginning of the school day and after lunch at British schools when students attend a short class; their form teacher marks attendance and gives out school notices. Form teachers are teachers of any subject assigned to a tutor group with responsibility for their pastoral care. Form teachers are usually assigned to a form group in year 7 and stay with that same group until the students leave school at the end of year 11.

### Measurements

Measurement sessions occurred 8 weeks apart; the first before the GoActive intervention started and the second during the final week of the GoActive intervention. All measurements occurred at both measurement sessions.

#### Accelerometry—primary outcome

At the end of the measurement session, participants were asked to wear an accelerometer (ActiGraph GT1M or GT3X) for 7 days before collection the following week. An explanation regarding monitor use was given, as well as an information sheet for participants. The ActiGraph has been shown to accurately assess energy expenditure among European adolescents during free-living conditions.^15^ ^16^ The monitor was set to record vertical acceleration at 5 s epochs. Participants were asked to wear the monitors during waking hours for 7 days and to only remove them for water-based activities. Owing to resource constraints, not all participants could be offered an accelerometer; participants were randomly invited to wear a monitor with random numbers used to decide allocation prior to the measurement session. Participants wearing a monitor at baseline were first allocated a monitor at follow-up; remaining monitors were then allocated on a first come, first served basis to the remaining participants. Moreover, participants who had not returned a monitor from baseline were not invited to wear another at follow-up. After returning their monitors after the second measurement session, participants were offered a £10 Amazon voucher.

Accelerometry data were analysed using a batch processing programme (ActiLife) to remove periods of ≥60 min of continuous zeros^17–19^ which were classified as non-wear time.^20^ The first (partial) day of measurement was not used for analysis. All participants with at least 1 day of at least 500 min of measured monitor wear time between 6:00 and midnight were included in this analysis. Cut-points^21^ were used to estimate moderate-to-vigorous physical activity (MVPA; ≥2000 counts/min) which have been used previously.^22^ We aimed to assess feasibility of accelerometry for potential future evaluations of this programme, especially considering the short time between premeasurements and postmeasurements. Owing to only being able to assess a subsample of participants, these data were used to assess compliance to measurements and acceptability of repeated monitor wear rather than as an assessment of PA level.

#### Questionnaire—secondary outcomes

Participants were asked to answer a questionnaire to assess the acceptability of questions, the length of the questionnaire and the feasibility of conducting all measures in one school lesson. The same questions were used for the pilot study and are described below.

#### Anthropometry

Researchers used standardised protocols to measure height and weight. Height was measured to the nearest millimetre (Leicester height measure, Chasmors, Leicester, UK). A non-segmental bioimpedance scale was used to measure weight (to the nearest 0.1 kg) and impedance in light clothing (Tanita, type TBF-300A, Tokyo, Japan). Height and weight were used to calculate body mass index (kg/m^2^). Weight status was derived using sex-dependent and age-dependent cut-points.^23^ Previously validated and published equations were used to calculate body fat percentage (BF%).^24^ Age and gender were self-reported. Anthropometric data were used descriptively (table 2).

**Table 2:** Descriptive characteristics of participants in feasibility study and pilot randomised controlled trial

		Pilot CRCT	
	Feasibility study	Control	Intervention
N schools	1	1	2
N participants invited*	234	138	458
N parent opt out	9	6	23
N student opt out	13	0	8
N non-attendance	29	17	82
N assented	183	115	345
N 2 waves measured	160*	115	285
N 2 waves AG	57	68	152
N 2 waves ≥3d AG	52	43	112
Age	13.7 (0.4)	13.1 (0.3)	13.2 (0.4)
Sex N (% male)	71 (43.3%)	50 (43.5)	164 (47.7%)
Height (cm)	165.8 (8.8)	161.8 (7.0)	162.6 (8.5)
Weight (kg)	58.7 (12.7)	53.0 (10.6)	53.4 (10.6)
BMI z-score	0.63 (1.2)	0.52 (1.1)	0.44 (1.1)
Per cent of overweight/obese	26.9	22.7	24.1

Mean (SD) unless otherwise stated.

*Not all participants given accelerometer; 113 participants at baseline, 123 at follow-up and 87 at both baseline and follow-up.

AG, actigraph; BMI, body mass index; CRCT, cluster-randomised controlled trial.

### Process evaluation

Participants were asked via questionnaire whether they were willing to be contacted to take part in a focus group about the acceptability of GoActive. We conducted six focus groups of between three and nine participants. These focus groups took place during school time and followed a topic guide. They were recorded and transcribed verbatim and transcriptions were made anonymous.

Owing to the need to make improvements to the programme before continuing with the pilot CRCT within a short timeline, it was not possible to use a coding process with transcribed data from the focus groups before making programme changes. However, three researchers independently read transcripts (KC, HEB, AS) and highlighted quotes which related to potential programme or measurement improvement. Initially highlighted quotes were used to derive broad themes and relevant data extracts were collated within the identified themes.^25^ After finalising themes, the contents were discussed, interpreted and summarised and example quotes selected to represent wider views and are presented in table 3.

**Table 3:** Summary of changes made to the GoActive intervention and study design between feasibility and pilot studies and changes still required after the pilot study with supporting information

Intervention			
Issue from feasibility study	Improvements (between feasibility and pilot)	Changes required after pilot	Supporting quotes from student focus groups
*Lack of mentors* Mentors were not recruited as they had examinations.	We emphasised the importance of the mentors to the pilot schools at recruitment. Mentors were successfully recruited in one of two intervention schools during the pilot study.	Reiterate importance of mentors at school recruitment Participating schools to sign a contract agreeing to recruit mentors Regular contact with schools during planning to confirm mentor recruitment Recruitment two terms before intervention beginning to allow schools planning time	“…so for instance a sixth former came into our form and we was not very motivated, didn't really want to do it and he's in there saying, right, we're all going to go outside, we're all going to do this, I think probably, I don't know, I'd probably give it more effort…” Male participant (postfeasibility focus group) “Mentors would have been helpful especially with large tutor groups.” Teacher (postpilot questionnaire)
*Lack of clarity at start* Researchers did a launch assembly at the beginning of the project but students suggested the need for clearer initial intervention explanation.	Mentors provided initial support at one school. One hour mentor training was conducted prior to intervention start with emphasis on teacher training. Ongoing support for mentors and teachers was provided by facilitators.	Video explaining the intervention Video explaining the difference between participation in measurements and the intervention Videos of included activities Full day mentor training	“It was just difficult to get them started but once they were into it it was fine.” Year 11 mentor (postpilot) “Not very sure what was going on, so form [teachers] looked disorganised.” Teacher (postpilot)
*Points recording complicated* The students found the system for recording points on ‘points-cards’ too complicated; this was also a burden for study staff entering the points.	*Simplified points entry system* Simplified points system Simplified recording system Initial development of website functionality to allow online points entry by participants	Website to allow online points entry Participants, mentors and teachers can upload points. Facilitator will be able to track points entry and issue reminders.	“They [pointscards] were like complicated, there was too many like days and numbers and you didn't know where to like put it.” Female participant (postfeasibility)
*Activity preferences* Participant focus groups revealed occasional sex imbalance in activity choices, and with that differential motivation to participate.	*Boy and girl leaders each week* One boy and one girl in each form to be leaders each week to ensure a range of activities	At the intervention mid-point schools will be encouraged to add additional activities to maintain the novelty aspect of the intervention. Mentor training will include importance of varied activity selection.	“Yeah, like our sports is for what like the leaders want to do, not the whole class, ‘cos all the boys would pick like boxing and the girls want to do like dancing and Zumba but the boys don't want to do that so we all go for the boys one, but ‘cos we have a girl and a boy we should like the boys do their thing and the girls do their thing with their leaders.” Female year 9 participant (postfeasibility)
Continued			
**Table 3** Continued			
Study design			
Issue	Proposed change		Supporting information
*Questionnaires* Some students had difficulty completing questionnaires.	*Word substitutions and font/colour change* Word substitutions and explanations added (eg, optimistic changed to hopeful) Questionnaires to be printed on coloured paper to help students with learning needs	We will additionally assess group cohesion and social networks to further elucidate potential mechanisms of the intervention.	Informed by teachers’ suggestions during measurement sessions Rationale for adding additional questions: 44% of pilot participants stated that they asked someone to do physical activity with them during the intervention.
*Measurement session attendance* 12.4% of eligible students in feasibility study did not attend a measurement session due to absences, illnesses, forgetfulness and apathy.	Measurements were conducted on more than 1 day where possible.	Encourage contact teacher to locate pupils during measurements Multiple measurement days per school Aim for one consistent member of project staff to build a relationship over time with two contact teachers	In pilot non-attendance (% excluding opt-outs) varied: 8.0% helpful teacher with 1 measurement day 17.6% non-helpful teacher with 2 measurement days 20.7% non-helpful teacher with 1 measurement day
*Measurement incentives* Students did not realise that they were receiving vouchers for participating in measurements in feasibility study.	*No monetary incentives* Used low cost gifts in the pilot trial as the feasibility school were not enthusiastic about the vouchers (∼20% students eligible for free school meals)	No further changes	Recruitment and retention was similar in feasibility study and pilot trial
*Accelerometer data* Not all participants could be issued an accelerometer due to resource limitations but 6% monitors were lost	*Strategies for monitor return* Teachers and mentors were asked to remind students to return monitors During measurement sessions, more emphasis was given to monitor explanations and the importance of wear and return	Email reminders to students during the measurement period and prior to monitor collection During accelerometer fitting graphs of wear and non-wear will be shown Form teachers will be given lists of students not returning monitors	Pilot study return rate and compliance needs improvement; 36.9% students returned two waves of valid accelerometer data and across three schools monitor losses were 8%, 3% and 3%

Form teachers were asked to complete a questionnaire after the intervention had finished. This asked whether the teachers enjoyed the programme, whether it was fun for the class, whether they thought it made their class more active, whether it was a lot of work and whether the students found it boring; all items had response categories from 1 (strongly agree) to 4 (strongly disagree). Teachers were also asked to write free-text comments regarding suggested improvements.

## Statistical analysis

Anthropometric and PA data from the feasibility study are presented descriptively.

## Results

The intervention was delivered by the school to the whole of year 9 with limited researcher assistance for 8 weeks during Summer term 2013. Despite initial agreement, the school was unable to provide mentors as it was Summer term and the older students had examinations. Year 9 form teachers were trained to deliver the intervention prior to the programme starting; the teachers delivered the intervention with the help of one GoActive team member (KC or AS) during tutor time once per week. A total of 234 year 9 students were exposed to the intervention as reported by the school (N=234) with 9 parents (3.8% of eligible students) and 13 (5.6%) students opting out of participation in study measures. A total of N=183 (78.2%) assented to participate in measurements with 29 (12.4%) not attending a measurement session (eg, due to absence or apathy).

Participants were mean (SD) 13.7 (0.4) years old, 43.3% male and 26.9% were overweight or obese. Participants liked wearing the monitors and although only 113 participants were able to wear a monitor at baseline, 123 participants wore an accelerometer at follow-up and demand exceeded availability. Of the 87 participants who wore an Actigraph at both baseline and follow-up, 66% and 60% returned ≥1 and ≥3 valid days of data, respectively. Unfortunately a school trip on the postintervention measurement day meant that some participants who wore a monitor at baseline were unable to be assessed at follow-up; this is rationale for introducing multiple measurement days per wave per school. Participant information is presented in table 2.

### Process evaluation

Student quotes have been selected where relevant to support the suggested programme changes, prior to the pilot trial, as summarised in table 3. In brief, the main changes required between the feasibility and pilot trial regarding the intervention were identified as (1) the need for mentors, (2) better initial support and training, (3) a simplified points system and (4) a boy and a girl in-class peer leader each week. Regarding measurements, the needs identified included word substitutions and font/colour change for improved questionnaire completion, multiple measurement sessions per school, no monetary incentives and multiple strategies for monitor return.

Of 9 eligible form teachers involved in the project, 8 completed questionnaires; 7/8 teachers enjoyed the programme, 7/8 thought that their class did more activity, 6/8 thought that their class found it fun, 3/8 thought it was a lot of work and only 2/8 thought that their class found it boring. Most of the free-text comments highlighted the need for improved organisation and information provision at the beginning of the project and confirmed the importance of mentors. Teacher suggestions are included in table 3 where relevant.

## Feasibility study discussion

We were successful in recruiting and consenting 78.2% of a year group to measurements and delivering the intervention to the whole year group. Although only 9 parents opted their son/daughter out of measurements and 13 students did not assent to measurements, 29 (12.4%) of eligible participants did not attend a measurement session due to school-reported absences, illnesses, forgetfulness and apathy. Clear pointers for improvement were identified based on feedback from schools, teachers, students and our process evaluation data. These suggested improvements related to the intervention and also to the measurement sessions and highlight the value of a feasibility study of an intervention programme and evaluation methods irrespective of the previous research experience of the team. The changes required between the feasibility and pilot stages of this project are described in table 3 and are presented as broad themes in this discussion to avoid repetition.

Many of the improvements needed regarding the intervention relate to communication and training between the research team and the school. These issues were relatively difficult to address and warranted further piloting to improve various elements of the programme and evaluation. We were surprised by how difficult it was to recruit mentors given that the school was initially very keen on this element of the programme; we hoped that running the intervention in a school term without examinations might be more successful. Also, despite running a training session for form teachers, not all attended and it was difficult to gain contact to the other teachers in order to convey the salient information. We were able to run the programme in all year 9 tutor groups but it took a few weeks of research team efforts to get some of the classes fully understanding and participating.

Suggested changes to the measurement methods were mainly operational and theoretically relatively easily addressed as they are mainly regarding logistics of study conduct. However, some suggestions such as organising different days of measurement sessions at each school require collaboration from the school and may prove more challenging.

The majority of the changes required are either surrounding the need for improved communication between the research team and the school and second aligning initial promises by schools with what they are able to operationalise in practice.

## Pilot randomised controlled trial

In Autumn term 2013/Spring term 2014, we conducted a *cluster-randomised controlled pilot trial* in three schools (two intervention schools; ISRCTN31583496). The aim was to assess preliminary effectiveness and estimate the number of participants required to adequately power a full trial, to test measurement logistics, the feasibility of randomising schools and training intervention facilitators outside of the research team.

## Methods

### Recruitment and randomisation

School recruitment, participant recruitment and consent procedures followed the process outlined for the feasibility study. All non-fee-paying (state), all-ability secondary schools within a 30 min drive of the study office were sent a letter inviting them to take part; the first three to agree were included. Following successful recruitment of three schools, recruitment of the remaining schools was no longer pursued. After recruitment, randomisation was conducted using random number generation by an individual outside of the research team. The control school was offered (but did not take) the full GoActive programme materials and preprogramme training after completion of follow-up study measurements.

### Mentor recruitment

Schools were asked to recruit two older students per year 9 form to act as mentors; as mentorship involves a time commitment and a particular skillset (eg, able to lead year 9 and motivational individuals), we considered that it was most appropriate for schools to nominate students. After recruitment by the intervention schools, they were to be provided with written information regarding the study. A 1 hour training session was then given to mentors by the study team prior to the start of the intervention and the mentors received ongoing support from the intervention facilitators.

### Intervention delivery

The intervention was delivered to the whole of year 9 in both intervention schools. One school had ‘vertical forms’ where tutor groups consisted of students in every year group in the school. GoActive was adapted accordingly with all forms (and therefore all age groups) participating in the GoActive activities with year 9 students attending measurement sessions and recording points. We had agreed with the school that mentors were to work across house groups rather than in individual forms; however, the school did not use mentors to deliver the intervention; instead form teachers filled this role. In the other intervention school (which had a traditional form structure), mentors were recruited and facilitators outside the research team worked with them as planned to deliver the intervention to the year 9 forms.

### Measurements

Measurements occurred using the same format as the feasibility study; all measurements were conducted at baseline and 6–8 weeks after baseline (while the intervention was running) and where possible multiple measurement sessions were conducted at each school to enable us to measure participants who were absent on the day of measurement, who forgot to attend or who did not want to attend initially who changed their mind.

#### Accelerometer—primary outcome

PA data were collected and summarised as described above, although all participants were asked to wear an accelerometer at baseline and follow-up. Participants received a GoActive pen after the first measurement session and a choice of GoActive gift after completing the final set of measurements and returning their accelerometer (eg, Frisbee, bag, sports water bottle).

#### Questionnaire data—secondary outcomes

Questionnaire data were collected at baseline and follow-up. PA type was assessed using the 30-item Youth Physical Activity Questionnaire (YPAQ), which has been used in the same way previously among 13–14 years old.^26^ Participants were asked to state whether they had participated in any of the listed activities in the previous week with options to add extra activities; the number of activities reported was summed for each participant. To assess self-efficacy in support seeking,^27^ the participant answered yes (1) or no (0) to: *I can ask my parent to: sign me up for PA; my parent to do PA with me; my best friend to do something active with me* and a summed score was used. For social support for PA^28^ the participants answered yes (1) or no (0) to: *During a typical week, do the following things happen: my friends do PA with me; I ask friends to do PA with me; my friends ask me to do PA with them* and responses were summed. Further items included friendship quality which assessed eight items on current friendship satisfaction such as happiness with number of friends;^29^ item responses were summed with a higher value representing a more positive score. Well-being was assessed using the Warwick-Edinburgh Wellbeing scale with 14 positively worded items,^30^ each item had responses on a five-item scale (none of the time to all of the time) and responses were summed with higher scores representing higher well-being. Shyness and sociability were assessed with two five-item measures from the Emotionality, Activity, Shyness and Sociability (EAS) temperament scale;^31^ ^32^ each item was ranked by participants from 1 ‘not typical’ to 5 ‘very typical’; questions included ‘I make friends easily’ (shyness) and ‘I like to be with people’ (sociability); items were summed, so higher scores indicated lower shyness and higher sociability. To assess personal barriers to participating in PA, the participants answered yes (1) or no (0) to: *Are you ever stopped from doing PA because: there you want to watch TV; you don't think you're good at PA; you don't like PA; and you might get hurt* and responses were summed.

For descriptive purposes, anthropometric data were collected as described for the feasibility study. The primary outcome was min/day of MVPA; self-reported data were secondary outcomes.

## Statistical analysis

Analyses were performed using STATA V.14.0 (Statacorp, College Station, Texas, USA).

The primary outcome, MVPA, at baseline and follow-up was compared between intervention and control groups using analysis of covariance, with adjustment for baseline MVPA and change in monitor worn time (follow-up minus baseline). The same process was used to examine secondary outcomes (self-reported outcomes). There were not sufficient clusters to be able to adjust for school clustering in the analysis; results should therefore be interpreted with caution. The researchers conducting accelerometer processing were unaware of the intervention condition of participants.

## Process evaluation

We invited all intervention participants and mentors to complete a brief questionnaire about their experiences of the programme. Mentors provided written consent for participation in process evaluation; for mentors under 16, their parents provided informed passive consent and they provided written assent.

### Questionnaires

Year 9 participants were asked whether GoActive was fun, whether it encouraged them to do more PA, whether it increased confidence and whether they will continue with an activity they tried during GoActive after the programme. Participants who acted as year 9 peer leaders and the older mentors were asked whether GoActive was fun, whether they thought that it improved their leadership skills and whether it took up a lot of time. All items were scored on a four-point scale of strongly agree, slightly agree, slightly disagree and strongly disagree which were dichotomised as agree and disagree.

### Focus groups

We conducted two mentor focus groups during school time following a topic guide; each focus group included six participants. We also conducted a focus group with the two intervention facilitators after completion of the intervention. Unfortunately we were unable to conduct a focus group with year 9 students after the pilot study. These focus groups were recorded, transcribed and transcriptions were made anonymous, so that no participants could be identified from them. Using the method described for the feasibility study focus groups, the project team (KC, AS, HEB) recorded the points for improvement prior to progression to a fully powered randomised controlled trial. Teachers were asked to complete the same questionnaire as in the feasibility study.

## Results

**Figure 2:**
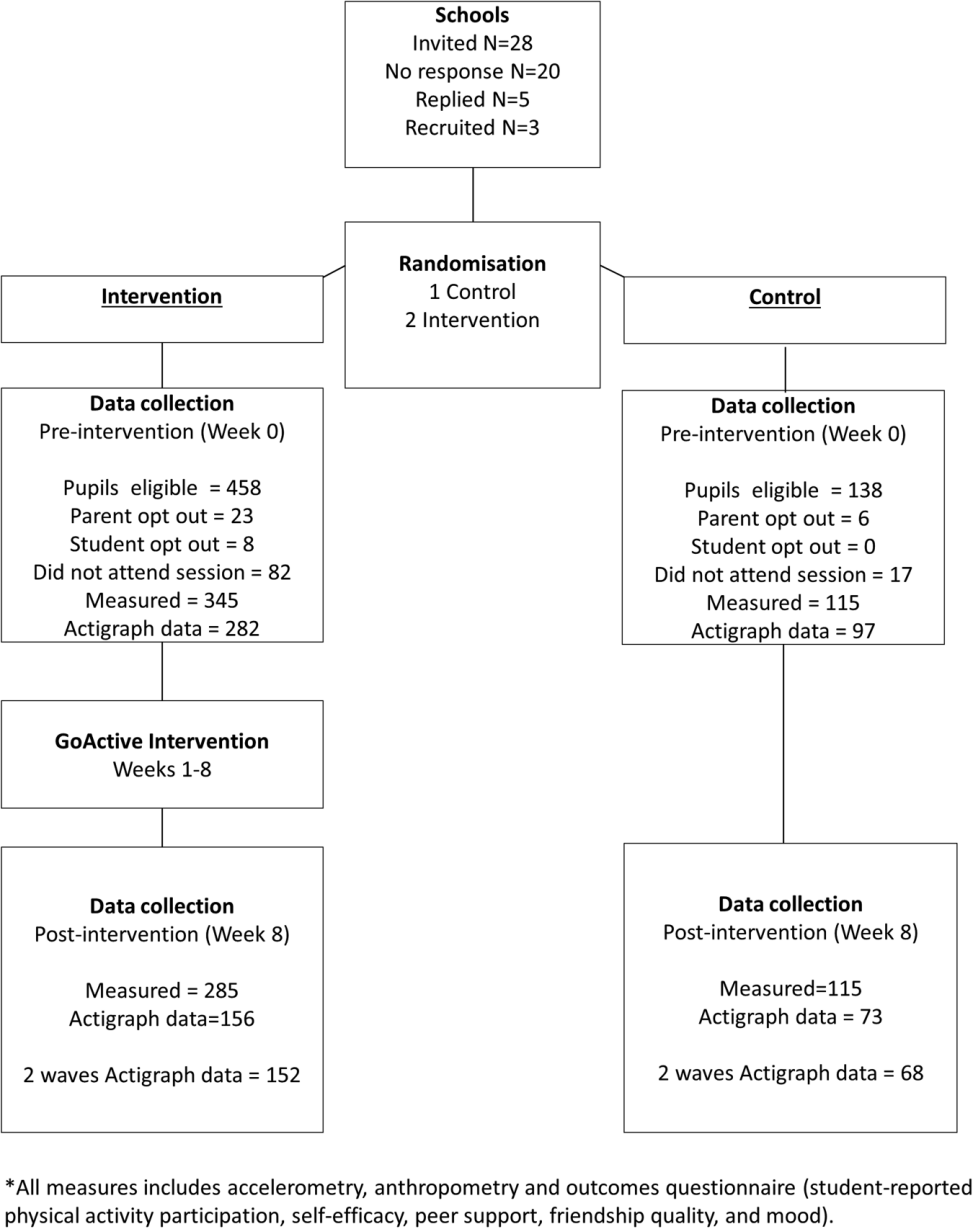
Pilot study recruitment flow chart.

Participation in the pilot CRCT is outlined in figure 2 and descriptive characteristics are presented in table 2. Across the three pilot schools, 596 year 9 students were invited to participate in the evaluation of GoActive; 458 provided valid written consent and were measured (76.8% response rate, average N=153 per school). Non-response was due to parental opt-out (N=29, 4.9%), student opt-out (N=8; 1.3%) and non-attendance of measurement sessions (N=99; 16.6%). Intervention and control participants were mean (SD) 13.2 (0.4) and 13.1 (0.3) years old, 47.7% and 43.5% male, and 24.1% and 22.7% overweight and obese, respectively.

Of 458 baseline participants, 87.3% attended the follow-up measurement; of these 400, 55% were available for analysis of the primary outcome (N=220 (≥1 day of ActiGraph data at pre and post)) and all 400 completed questionnaire-based measures assessing secondary outcomes. Average days of accelerometer wear were 4.9 (1.8) days preintervention and 3.8 (1.8) days at the second measurement; during those days average wear time was 776.6 (97.1) and 758.0 (103.3) min/day.

The results of this pilot CRCT provide an indication of the potential effect of GoActive on the main outcome measure; average daily minutes in MVPA (table 4). Change in MVPA in the control group was −6.5 (14.0) min/day and −2.5 (15.4) min/day in the intervention group with change adjusted for baseline 5.1 (1.1 to 9.2) min/day in favour of the intervention group. Further, the results of the questionnaire-based measures indicated tentative positive effects for some secondary outcomes including well-being and social support (table 5). However, as this was a pilot CRCT with only three schools, we were not able to adjust for school clustering and this pilot CRCT was not adequately powered to establish effectiveness. Owing to this small number of clusters, we would not necessarily expect intervention and control groups to be similar at baseline.

**Table 4:** Average daily minutes in MVPA by study group at baseline and postintervention, and preliminary intervention effect of GoActive pilot trial

	Control (SD)	Intervention (SD)	Difference adjusted for baseline (95% CI)
Feasibility study			
MVPA (baseline)		60.7 (27.5)	
MVPA (postintervention)		61.3 (25.6)	
Pilot trial			
MVPA (baseline)	48.6 (15.4)	51.9 (15.3)	
MVPA (postintervention)	42.1 (15.0)	49.4 (18.2)	
MVPA (change)	−6.5 (14.0)	−2.5 (15.4)	5.1 (1.1 to 9.2) p=0.014

*School-level clustering not taken into account due to insufficient clusters.

MVPA, minutes in moderate-to-vigorous physical activity.

**Table 5:** Secondary outcomes at baseline and postintervention; results show change adjusted for baseline

	Control (SD)		Intervention (SD)		
	Baseline	Follow-up	Baseline	Follow-up	Difference adjusted for baseline (95% CI)
Types of PA	19.2 (12.8)	14.0 (9.4)	19.8 (15.2)	16.6 (14.0)	2.3 (−0.2 to 4.7) p=0.07
Self-efficacy for PA	17.7 (0.4)	17.2 (3.6)	17.8 (3.0)	17.6 (3.2)	0.3 (−0.4 to 1.0) p=0.36
Peer support	6.3 (2.6)	5.3 (1.9)	5.9 (2.2)	5.5 (2.2)	0.5 (0.1 to 0.9) p=0.03
Friendships	2.8 (1.1)	2.9 (1.0)	2.8 (1.1)	2.9 (1.1)	−0.1 (−0.3 to 0.1) p=0.37
Well-being	44.5 (0.9)	43.3 (1.0)	45.0 (0.5)	45.5 (0.5)	1.8 (0.1 to 3.4) p=0.04
Shyness	13.9 (3.5)	14.0 (3.7)	13.7 (3.4)	13.7 (3.3)	−0.3 (−0.9 to 0.4) p=0.43
Sociability	13.5 (2.0)	13.9 (1.9)	13.7 (2.1)	14.0 (1.8)	0.1 (−0.4 to 0.5) p=0.74
Barriers to PA	29.7 (5.1)	28.7 (5.3)	29.1 (5.2)	28.4 (5.4)	0.1 (−1.1 to 1.2) p=0.91

Analyses not clustered for school as insufficient clusters.

PA, physical activity.

## Process evaluation

### Year 9 participants

Questionnaire data showed that for boys and girls, respectively, 71% and 74% agreed that taking part in the intervention was ‘fun’ and 56% and 69% said that it encouraged them to do more activity. Moreover, 61% of intervention participants indicated it fairly likely that they would continue with an activity they had tried during GoActive (64% boys, 59% girls). Of those who had been involved as peer leaders, 81% reported that they thought that was ‘fun’, 54% said that it had ‘improved their leadership skills’ and 38% said that it took up a lot of time.

### Mentors

In focus groups, mentors indicated that although they found it difficult ‘to get their head around’ the GoActive intervention, they quickly picked it up and enjoyed it (table 3). Out of 16 mentors completing a questionnaire (16 mentors invited), 14/16 (88%) agreed that GoActive was fun, 15/16 (94%) agreed that it improved leadership skills and 4/16 (25%) said that it took up a lot of time. Useful suggestions for improvements were made regarding the need for refined points collection, more comprehensive activity explanations, the importance of teacher involvement and more initial training which will be incorporated in the full trial and is summarised in table 3.

### Teachers

The school with vertical forms had year 9 students spread over all 66 school forms whereas the other school had a traditional form structure with eight year 9 forms; 11 teachers completed the questionnaire consisting of 5/8 (63%) from the traditional school and 6/66 (9%) from the vertical school. Across both schools 10/11 (91%) teachers enjoyed the programme, 8/11 (73%) thought that their class did more activity, 11/11 (100%) thought that their class found it fun, 2/11 (18%) thought it was a lot of work and none (0%) thought that their class found it boring. Similar to the feasibility study, most of the free-text comments highlighted the need for improved information provision between the research team and the school. Teacher suggestions are included in table 3 where relevant.

## Pilot CRCT discussion

We successfully tested measurement logistics, randomisation, trained intervention delivers outside the research team, ran the intervention in two schools and established preliminary effectiveness of the GoActive programme. Although the programme was improved compared with the feasibility study, the programme and evaluation methods still could benefit from further improvements. We used information from measurements, staff feedback, mentor and facilitator focus groups, and teacher questionnaires to iteratively improve the programme and evaluation. The changes required between the pilot study and a full effectiveness trial of GoActive are described in table 3 and are presented as broad themes in this discussion to avoid repetition.

Based on the pilot results, in a full trial, we would aim to detect a 5 min difference in MVPA (min/day). A 5 min increase is relevant at population level as it would increase the proportion of adolescents meeting the guidelines of 60 min of MVPA per day from 43% to 50% (based on baseline pilot data), with potential to significantly impact on population health.^33^ Based on this pilot data, we estimate N=1310 participants will be required for the primary effect analysis in a full trial. However, due to our low monitor compliance (39% in pilot trial) and to account for potential school drop-out, we aim to recruit 16 schools with 150 participants each (total N=2400; average recruitment per school in pilot=153). We have based these estimations on 30–40% lost to follow-up as we are confident that our changes will improve monitor compliance in future. The levels of MVPA are comparable to previous assessments in 13–14 years old British adolescents.^34^ The MVPA of intervention and control groups decreased; taken together with other evidence showing declines of MVPA during adolescence,^35^ adolescent PA promotion strategies may be valuable if preventing a decline even if not managing to increase MVPA. It was a limitation of this pilot CRCT that we only included one control school; this was partly due to time and resource restrictions for this pilot phase of research. However, including one control school allowed us to meet the main aims of our pilot CRCT of assessing trial logistics (including randomisation of schools) and estimating preliminary effectiveness. We were able to use data on school-level variability in MVPA from a previous study across multiple secondary schools to incorporate in our power calculations for the full trial.^34^

In the pilot CRCT we recruited mentors in one intervention school but not the other. From this, we learnt the importance of continued communication with school contact teachers and aligning initial promises by schools with what they are able to operationalise in practice. Issues surrounding communication still require improvement and show the need to streamline information for mentors, teachers and students to ensure it is comprehensive and consistent. In future, we plan to do this through videos explaining the evaluation and the intervention programme and also with individual activity videos for use during tutor time.

## Overall discussion

We aimed to assess the feasibility of study procedures and the implementation of the GoActive intervention across the whole of year 9, and to estimate preliminary effectiveness. Further, we aimed to estimate the number of participants required to adequately power a full trial to assess the effectiveness of the GoActive intervention to increase MVPA among 13–14 years old adolescents. We successfully ran the programme in three schools and assessed preliminary effectiveness, allowing for drop-out we would need to recruit 16 schools with 150 participants each (total N=2400) for a full trial.

### Improving participant retention

We used parental opt-out consent in the research reported here and found that our initial recruitment rates over the feasibility (78%) and pilot trial (77%) using this strategy were substantially higher than our previous UK-based research in this age group using parental opt-in consent (23% of eligible participants).^1^ However, despite high recruitment and retention, the number of participants available for analysis of the main outcome was lower than expected, predominantly due to difficulties with monitor wear and return at follow-up. This was irrespective of our liberal inclusion criteria of including all participants with at least one valid day of data; limiting the ability of these results to be representative of habitual activity. After speaking to participating schools and students, and with other investigators, in the full trial, we will aim to use various methods to improve monitor wear compliance and return such as increased emphasis on the importance of wear and return during the measurement session, multiple reminders to wear monitors during the measurement period, and teacher assistance. Obtaining parental opt-out consent has enabled us to recruit a higher proportion of the sample, but comes with drawbacks. This includes that we do not have access to parent or student mobile phone numbers so cannot provide reminders via text messages. However, we will aim for a key member of project staff to build good relationships with two key staff members from each school during the project to help improve communication, and with that, accelerometer wear and return rates.

### Increasing emphasis on mentoring

The experience of conducting the feasibility and pilot trial resulted in multiple lessons learnt and subsequent improvements to the intervention design at each stage of the project. Improvements between the feasibility and pilot study focused on a greater emphasis on mentorship, training of mentors and staff, streamlined recording of intervention points and standardisation of intervention delivery. We were surprised by the difficulty in recruiting and training mentors, despite schools liking this element of the programme and leadership training already being common at secondary schools. We had no success in recruiting mentors in the feasibility study and although we were successful in recruiting mentors for the pilot trial in one school, mentor feedback suggested that more thorough training and support was necessary prior to intervention initiation. Rather than a 1-hour training session as conducted for the pilot CRCT, we plan a full day session which will hopefully alleviate these issues and provide a stronger basis for the intervention. In one pilot school, we were unsuccessful contacting mentors despite promises from the school. This highlights the need to keep in regular contact with the contact teacher and to confirm that intervention steps have been completed prior to the intervention beginning. These issues were likely exacerbated by the short time frame in which we had to recruit schools and begin the intervention. Teachers told us that it would be easier with a longer lead time for schools; therefore, the full trial allows recruitment two terms prior to the intervention starting to allow for sufficient preparation, mentor recruitment, and training for teachers and mentors. Although there are clearly challenges with mentors (15–18 years old) being expected to deliver the bulk of the intervention, this is an increasingly popular strategy in health promotion research^36^ ^37^ and means that programmes are potentially more cost-effective and sustainable. To further support the mentors through the initial weeks of the intervention, we will allocate an externally funded facilitator half a day per week to each intervention school.

### Developing the intervention website

Mentors and teachers found supporting students recording points challenging so funding is allocated within the full trial budget to enable further development of the website platform to enable electronic submission and tracking of points. Further, we plan for this website to contain sufficient information for a school to run GoActive independently which could facilitate potential future use of the intervention with limited outside support. An information video will also be produced which will explain the difference between intervention and control conditions and provide a brief explanation of the GoActive intervention for use at the beginning of the study to ensure consistency of explanation. This will also allow mentors and teachers to remind themselves of the process during the challenging initial phases of the project.

### Refining measurement sessions

Our process evaluation and focus groups also provided insight into how we could improve the study design in general, including the measurement sessions. We believe that this type of information, while rarely published, is valuable to the progression of the GoActive study but also for other researchers assessing PA at secondary schools. This information included the organisation of more than one measurement session per school at each time point as non-attendance on this day may influence recruitment and retention. Further, as suggested by teachers, we will print questionnaires on coloured paper, in at least size 12 font without serifs to help students with reading difficulties. Our secondary outcomes indicated no evidence of harm but we will continue to monitor any potentially adverse events in future work.

### Incentivising teachers

It was noticeable from some of the student focus groups during the feasibility study that the enthusiasm of the teacher was important for adherence to the intervention; students were more positive about the intervention when the form teacher was really invested in the programme. This was highlighted when a participant who initially did not record points moved forms and stated in the focus groups how much he liked the programme, and had participated when motivated by his new class. We plan to incentivise teachers in intervention schools by giving small gifts at the end of the study for those whose forms engage. To further standardise intervention delivery and provide a consistent element of the intervention across schools, we aim to develop activity videos to be used. This was suggested by a teacher to reduce burden of this intervention being delivered during tutor time in which other demands are placed on teachers' time.

While we collected valuable qualitative data during our participant and mentor focus groups, we did not have time to conduct formal qualitative analysis and we were also unable to conduct student focus groups after the pilot phase. These are limitations of this research but were necessary in order to progress the research at a timely pace, and to meet the timing of funding calls. However, it is important to use and publish this type of feasibility and pilot research as stated previously^38^ as often it is not properly used by researchers let alone published to enable use by others developing similar programmes. The nature of this formative research often requires long papers which may be difficult to publish. By combining feasibility, pilot and lessons learnt in one paper, we are hopefully highlighting the most useful and salient and messages without an excessive number of publications. We did not collect cost-effectiveness data in the feasibility and pilot studies and will put in place school-relevant mechanisms to collect the relevant data in the full trial.

## Conclusion

The feasibility study and pilot trial of the GoActive intervention showed feasibility of recruitment, measurement, randomisation and the ability to deliver GoActive to a whole school year group of 13–14 years old. Both of these stages prompted several key improvements to the intervention and to the study design including emphasis on monitor return, mentor recruitment, adequate mentor training, clearer and more consistent intervention explanations, and improved points recording systems. The lessons learnt from each phase of this research have been taken forward to an ongoing full trial to evaluate the effectiveness and cost-effectiveness of the GoActive intervention to increase MVPA among 13–14 years old.
